# Health worker views on pre-treatment loss to follow-up in adults with pulmonary TB in Western Kenya

**DOI:** 10.5588/pha.23.0016

**Published:** 2023-09-21

**Authors:** M. N. Mulaku, O. M. Corrie, I. Odero, T. Young, K. R. Steingart, E. Ochodo

**Affiliations:** 1 Centre for Global Health Research, Kenya Medical Research Institute, Kisumu, Kenya;; 2 Centre for Evidence-based Health Care, Division of Epidemiology and Biostatistics, Faculty of Medicine and Health Sciences, Stellenbosch University, Tygerberg, South Africa;; 3 Department of Pharmacology, Clinical Pharmacy, and Pharmacy Practice, Faculty of Health Sciences, University of Nairobi, Nairobi, Kenya;; 4 Department of Clinical Sciences, Liverpool School of Tropical Medicine, Liverpool, UK

**Keywords:** key informant interviews, healthcare system, qualitative

## Abstract

**SETTING:**

County referral hospital in Western Kenya.

**OBJECTIVES:**

To explore factors contributing to pre-treatment loss to follow-up (PTLFU) in adults with pulmonary TB and propose solutions to address PTLFU from healthcare worker (HCW) perspectives.

**DESIGN:**

This was an exploratory qualitative study using thematic analysis.

**RESULTS:**

We conducted 19 key informant interviews with HCWs representing laboratory, clinical care, management and the community. Participant age ranged from 26 to 62 years; 14 (74%) were females; and most (74%) had worked in TB care for ⩽5 years. They reported that patients experienced stigma and had misconceptions about TB that contributed to PTLFU. HCWs were hesitant to work in the TB clinic, which contributed to suboptimal patient care, leading to PTLFU. Unclear linkage between laboratory and clinician, and limited financial resources to track patients were among the healthcare system factors that led to PTLFU. HCWs suggested having proper patient preparation, assigning resources to track patients and holding regular interdisciplinary meetings as practical solutions to address PTLFU.

**CONCLUSION:**

HCWs reported multiple factors that may influence PTLFU and recommended various solutions to address these. Knowledge of TB management, patient preparation, resources to track patients and multidisciplinary meetings will be central to addressing PTLFU.

## Abstract (fr)

**CONTEXTE:**

Hôpital de référence du comté dans l’ouest du Kenya.

**OBJECTIFS:**

Explorer les facteurs contribuant à la perte de suivi avant traitement (PTLFU) chez les adultes atteints de TB pulmonaire et proposer des solutions pour traiter la PTLFU du point de vue des travailleurs de la santé (HCW).

**MÉTHODE:**

Il s’agit d’une étude qualitative exploratoire utilisant l’analyse thématique.

**RÉSULTATS:**

Nous avons mené 19 entretiens avec des informateurs clés représentant les laboratoires, les soins cliniques, la gestion et la communauté. Les participants étaient âgés de 26 à 62 ans, 14 (74%) étaient des femmes et la plupart (74%) travaillaient dans le domaine de la TB depuis ⩽5 ans. Ils ont indiqué que les patients étaient stigmatisés et avaient des idées fausses sur la TB, ce qui contribuait à la PTLFU. Les HCW hésitaient à travailler dans la clinique de la TB, ce qui a contribué à une prise en charge sous-optimale des patients, conduisant à la PTLFU. Le manque de clarté du lien entre le laboratoire et le clinicien et les ressources financières limitées pour suivre les patients font partie des facteurs du système de santé qui ont conduit à la PTLFU. Les HCW ont suggéré de préparer correctement les patients, d’affecter des ressources au suivi des patients et d’organiser des réunions interdisciplinaires régulières comme solutions pratiques pour remédier à la PTLFU.

**CONCLUSION:**

Les HCW ont fait état de multiples facteurs susceptibles d’influencer la PTLFU et ont recommandé diverses solutions pour y remédier. La connaissance de la prise en charge de la TB, la préparation des patients, les ressources pour suivre les patients et les réunions multidisciplinaires seront essentielles pour traiter la PTLFU.

Although preventable and curable, TB remains a top cause of death worldwide. The WHO estimates that, in 2021, 10.6 million people became ill with TB and 1.6 million people died.^1^ Kenya is among the top 30 high TB burden countries globally, with a national incidence rate of 251 persons/100,000 and mortality rates of 39 and 21/100,000 in HIV-negative and HIV-positive people, respectively, in 2021.^1^ Despite achieving the WHO End TB 2020 milestones,^2^,^3^ the continued high burden of TB indicates unidentified gaps in the continuum of care.

Pre-treatment loss to follow-up (PTLFU), i.e., when patients are lost to care after being diagnosed but not started on treatment, is an often-neglected component of TB care in high TB burden countries.^4^ Globally, PTLFU ranges from 4% to 38%, with the highest rates observed in Africa.^5^ Individuals with PTLFU have a higher risk of TB-related mortality and may transmit TB in the community,^5^–^7^ thereby undermining efforts to reduce the TB burden.^8^,^9^

Although little is known about PTLFU in Kenya, a cross-sectional study by Tollefson and colleagues on TB under-reporting suggests that PTLFU is an important concern. The researchers identified 3,409 TB patients with smear-positive sputum and found that one in five patients had not been reported to Kenya’s National Surveillance Programme and may not have started TB treatment.^10^ Furthermore, the study noted that the unreported TB patients were older, from large and high TB burden region facilities.

A systematic review by MacPherson and colleagues, including 23 studies, synthesised quantitative research on factors associated with PTLFU.^5^,^11^ Key patient-related factors were male sex, older age, living in an urban area, difficulty getting time off from work, and lack of understanding about TB. Healthcare system-related factors were long waiting times for health services, delays in receiving the results of sputum microscopy, geographical location of the TB laboratory and being diagnosed with smear-negative, culture-positive TB.^5^

Few studies, mainly conducted in India and South Africa, have used qualitative methods to give insight into how and why we lose people experiencing PTLFU.^11^ In these studies, patient reported factors placing people at risk for PTLFU included negative perceptions of healthcare worker (HCW) behaviour (having an offensive manner), poor communication following diagnosis and providing insufficient psychosocial support.^12^–^14^ Healthcare system-related factors contributing to PTLFU included frequent hospital visits, long waiting times, and human resource and financial constraints.^12^–^15^

To get a fuller understanding of PTLFU, we set out to explore factors contributing to PTLFU and propose solutions to address PTLFU from HCW perspectives in Kenya.

## METHODS

### Study population

We purposively selected HCWs at the TB clinic of Jaramogi Oginga Odinga Teaching and Referral Hospital (JOOTRH), Kisumu, Kenya. The purposive sampling took into consideration the principle of maximum variation where aspects of sex, age and cadre were observed. Likewise, one community health volunteer (CHV) involved in patient follow-up was purposively selected from each community unit attached to JOOTRH. We invited people by telephone or approached them in face-to-face meetings to participate in the study.

### Definition of pre-treatment loss to follow-up

We adopted the definition used by MacPherson et al.^5^,^14^,^16^ PTLFU was defined as patients identified by the national TB programme who received a diagnosis of pulmonary TB (PTB) based on at least one positive sputum smear or culture or WHO-recommended rapid diagnostic but did not start TB treatment within 14 days. This includes persons who died before initiating treatment.

### Study design

We used an exploratory qualitative design to understand the processes, experiences and perspectives of HCWs about PTLFU.

### Study setting

The study took place at JOOTRH Kisumu, Kenya. JOOTRH serves as the referral hospital for public, private and faith-based health facilities, with a catchment population of approximately 5 million people in over 10 counties in Western Kenya.^17^ The TB clinic at JOOTRH uses both paper and electronic records to manage patients with presumptive and confirmed PTB. JOOTRH currently uses Xpert® MTB/RIF (Cepheid, Sunnyvale, CA, USA) as the initial test to diagnose TB in all people with presumptive TB. Smear microscopy is used to monitor all TB patients with smear and Xpert-positive results on treatment at months 2, 5 and 6.^18^ Once a person with presumptive TB is identified, a sputum specimen is collected and sent to the JOOTRH main laboratory for Xpert testing. Patients are advised to return for their results the next day, but those who test positive for PTB are contacted by phone as soon as possible. Individuals who opt to receive care at the TB clinic are registered to start treatment, while those who prefer to receive treatment at another healthcare facility are given a 1-week treatment starter pack. JOOTRH has seven community units where CHVs can provide patient follow-up, supervised by community health extension workers.

### Data collection

MM and IO conducted key informant interviews in February and March 2022 at a convenient time and place for the HCWs. MM is a female epidemiologist and is well-versed in both quantitative and qualitative research methods. IO is a female social scientist, who has training in qualitative research methods and skills in conducting interviews. Before the interviews, we sought written informed consent from the HCWs to participate in and record the interviews. MM had an introductory session with all TB staff and informed them of the rationale, study aims and data collection methods. There were no pre-established relationships between the interviewers and study participants.

We developed an interview guide with questions that allowed the participants to narrate their understanding of and experiences with PTLFU, reasons for PTLFU and possible strategies to reduce PTLFU (Supplementary Data 1). We piloted the guide with two researchers by simulating an interview to understand the context, guide and flow of the interview questions. Thereafter, we refined the guide to ensure that the study aims were achieved through the questions. We conducted face-to-face interviews in English with HCWs which lasted approximately 25 min. The interviews were audio recorded and transcribed verbatim. In addition, we took field notes to describe participants’ behaviour and environment. Towards the end of the data collection period, data were saturated.

### Data management and analysis

We de-identified participants by ensuring their names were not used in the audio recording or the storage of the audio records. If names were mentioned during interviews, we removed them from the audio recording before transcription. We checked for accuracy of interview transcripts by listening to the recordings. Transcripts were not returned to participants for review or validation.

We used thematic analysis to analyse the data. MM and OMC read the transcripts several times for familiarisation before exporting them to QSR NVivo© 2020 (QSR International, Burlington, MA, USA)^19^ for data management and coding. We gave the participants unique numbers that were used to report their quotes, and we presented the quotes verbatim to ensure trustworthiness of our qualitative methods.^20^ We did not use cadres to report the quotes, as there was only a single participant in certain cadres, which could reveal their identity. We stored the audio files in a password-protected database and hard drive. MM developed a codebook where the codes and their descriptions were derived from the interview questions and existing literature. This was verified by OMC. MM and OMC applied the codebook to all interview transcripts independently. We then classified the codes into specific themes relevant to the research question. We held regular discussions to clarify differences in the application of the codebook and the identification of themes.

We summarised the findings into four themes: knowledge of and experience with PTLFU, extent of PTLFU, factors contributing to PTLFU and suggestions to minimise PTLFU. We further organised factors contributing to PTLFU according to the level where these factors were encountered: patient, provider and healthcare system.^21^,^22^

### Ethical considerations and reporting

Ethical approval was obtained from the study site’s ethics and review committee JOOTRH, Kisumu, Kenya (IERC/JOOTRH/563/21); Kenya Medical Research Institute, Kisumu, Kenya (KEMRI/SERU/CGHR/392/4309); and Stellenbosch University, Tygerberg, South Africa (S21/04/066(PhD)). We obtained written informed consent (Supplementary Data 2) and removed personal identifiers before data analysis. We reported the findings of this study following the consolidated criteria for reporting qualitative research (COREQ).^23^

## RESULTS

### Demographic characteristics of study participants

We conducted 19 key informant interviews with HCWs from clinical care, laboratory, management and community. The average age of participants was 40 years (range 26–62), and 14 (74%) were females. The average time in TB care was 5 years (range: 7 months–18 years) with 14 (74%) being in TB care for ⩽5 years (Table 1).

**TABLE 1:** Demographic characteristics of the study participants at Jaramogi Oginga Odinga Teaching and Referral Hospital, Kisumu, Kenya

Participant ID	Age (years)	Sex	Time in TB care (years)
KII001	53	F	2.5
KII002	31	F	0.6
KII003	29	M	6
KII004	30	F	5
KII005	27	F	7
KII006	32	F	1
KII007	53	F	3
KII008	31	F	1.5
KII009	27	F	2
KII010	62	M	5
KII011	50	F	12
KII012	60	F	5
KII013	55	F	3
KII014	50	F	5
KII015	46	M	18
KII016	37	F	10
KII017	26	F	1
KII018	29	M	1
KII019	42	M	12

F = female; M = male.

### Knowledge of and experience with pre-treatment loss to follow-up

Several participants correctly described PTLFU as a patient who had received a positive TB test result but was lost before starting treatment.

My understanding about PTLFU are these patients that we diagnose with TB and then before we start them on treatment then they are lost. (KII_004)

Others thought that PTLFU pertained to a patient who had received a positive TB test result but was lost after starting treatment.

I understand it as s/he has already been diagnosed and has started on drugs, and then all of a sudden s/he stops. Or s/he hides and goes home. (KII_007)

Few participants reported experience caring for people with PTLFU.

Yeah, we have had experiences with such cases. Because there are clients who come during the presumptive case-finding, then we do a GeneXpert. Then when we call them back, we don’t get them. Either we can get them on phone and they are not comfortable, then later on they switch off their phone. (KII_008)

### Extent of pre-treatment loss to follow-up

Participants reported that PTLFU was uncommon. Several participants reported observing only one or two people with PTLFU during the 3 months preceding the interviews.

I can’t say it is a very big issue with us. Because out of 50, we can have one pre-treatment loss to follow-up. (KII_008)

Nevertheless, one participant commented that even one person with PTLFU is a matter of concern, as it could lead to TB being spread to others.

It is a problem because if you test a client and he turns positive and he goes that way, this client is now spreading TB wherever he or she is. (KII_003)

In addition, participants thought that PTLFU was more likely to be found in high-volume health facilities in urban areas than in smaller facilities.

### Factors contributing to pre-treatment loss to follow-up

Supplementary Data 3 summarises reported factors influencing PTLFU at patient, provider and healthcare system levels.

#### Patient level

Participants mentioned that social issues, such as alcohol and drug use disorders (Q1, Q2) and lack of housing (Q3), influenced PTLFU. Participants noted that poverty contributed to PTLFU as some patients lacked enough money to buy food and pay for transportation to the health facility (Q4, Q5). Nearly half of the participants reported stigma (Q6, Q7). Other factors highlighted include misconceptions about TB (Q8), wrong contact details (Q9, Q10), religious beliefs (Q11) and living in a different geographical location from the hospital (Q12) (Supplementary Table S1).

#### Provider level

Participants shared that most HCWs were unwilling to work in the TB clinic (Q13, Q14). Other issues reported by HCWs leading to PTLFU were not keeping patient information confidential (Q15), poor attitude (Q16), not providing psychosocial counselling (support to address psychological, social, and economic factors that may prevent people from accessing care),^24^ and not giving patients enough time to adjust to their TB diagnosis (Q17, Q18) (Table 2).

**TABLE 2:** Representative quotes on provider-related factors contributing to pre-treatment loss to follow-up

Factor	Representative quote
Unwillingness to work in the TB clinic	*Q13: “What I have learned… what I have seen… when I went into TB, TB was kind of segregated personally when I came here clients were very sick, they were coming when they were initially very sick and also staff don’t like working in TB, that is what I would say. First, even me I didn’t like going there, I really bargained (took time to weigh the pros and cons about) my going there but I went there so we could find clients were coming and were very sick.” (KII_001)*
Lack of commitment	*Q14: “One of them I said is that commitment to TB management is still not 100%.” (KII_015)*
Breach of confidentiality	*Q15: “Confidentiality, I think. You know you should not share the secret of the patient with somebody. Maybe the patient is seated out there and hears the doctor tell the other patient, ‘You see this patient that is seated there, she’s like this and this.’ You know when the patient comes and tells you his/her problems, you should not tell the patient’s problems to somebody else.” (KII_005)*
Attitude	*Q16: “She will be knocking, and we are just seated, ‘What do you want?’ That is the first impression. ‘Sit there!’…we have a cough booth but someone is going to tell the patient, ‘Follow here and go and remove at the toilet. And hide so that nobody sees you.’” (KII_002)*
Lack of psychosocial counselling and support	*Q17: “Yes, sometimes even us as healthcare providers, there are parts where we miss it out. These are patients who need some psycho-social support before you tell them that they have been found positive and everything.” (KII_002)*
	*Q18: “Maybe related to a healthcare worker, maybe if you didn’t prepare that client in a proper way by creating a good rapport, such things could also lead to one not feeling comfortable of maybe understanding what he is suffering from that could lead to that person really not being eager to come for treatment.” (KII_004)*

#### Healthcare system level

Participants highlighted that there are no guidelines for PTLFU or staff assigned to follow-up on missing patients (Q19, Q20). Participants also pointed out the following issues leading to PTLFU: drug stockouts (Q21), long turnaround times for Xpert results (Q22, Q23) and unclear referral system from other facilities to the study site laboratory (Q24). Furthermore, lack of linkage from the laboratory to the clinician within the facility (Q25, Q26) and absence of a pharmacist in core TB team meetings may have contributed to disruption in the supply of TB medicines (Q27). Reliance on funding partners (Q28) and high staff turnover when there were changes in funding partners supporting the TB programme (Q29) were other factors that influenced PTLFU (Supplementary Table S2).

### Suggestions to reduce pre-treatment loss to follow-up

Participants suggested several practical solutions at the provider and healthcare system levels. At the provider level, participants proposed psychosocial counselling (Q30, Q31), good attitude towards people affected by TB (Q32, Q33) and proper patient preparation (Q34, Q35). At the healthcare system level, participants proposed carrying out TB awareness campaigns in the community (Q36), allocating more human and financial resources for TB services (Q37), using a locator form (Q38), involving CHVs in tracking missing patients (Q39) and providing an allowance to support CHVs doing the follow-up (Q40).

Additional strategies included having small groups where patients are supported financially for income-generating activities (Q41), informing patients about the nearest health facility offering TB treatment to mitigate travel costs (Q42), transmitting results to health providers electronically to decrease delays associated with laboratory reporting via email or telephone (Q43), involving pharmacists on the multidisciplinary team and having regular interdisciplinary meetings (Q44, Q45), providing training on TB and TB infection prevention and control (Q46), integrating TB services with those in other hospital departments to allow staff to rotate in the TB clinic (Q47) and ensuring a sufficient supply of laboratory materials and TB medicines (Q48) (Supplementary ­Table S3 and Supplementary Data 4).

## DISCUSSION

We interviewed 19 HCWs on contributing factors to PTLFU and actions that can be taken to reduce it. Factors contributing to PTLFU were identified at the patient, provider and healthcare system levels. Solutions proposed to reduce PTLFU were mainly directed at the provider and healthcare system levels.

HCWs were unwilling to work in the TB clinic because they feared becoming infected. This may have contributed to high workload of the staff available in the TB programme, which in turn, adversely affected the quality of patient care offered. Consequently, patients may have become discouraged from accessing further care. This aligns with a study conducted in South Africa.^13^ Providing HCWs with training to increase knowledge and skills to care for people affected by TB and training on TB infection prevention and control is essential to minimise the risks of acquiring TB.^25^,^26^ However, there is limited evidence to show the impact of training on TB patient outcomes.^27^

Lack of proper patient preparation was reported as a factor contributing to PTLFU. Having regular health talks with patients before they visit the clinic and offering psychosocial support could go a long way in giving them hope of becoming TB-free if medicines are taken as instructed.^28^ Psychosocial counselling could support patients, dismiss misconceptions they have concerning TB, and establish trust between patients and HCWs.^24^,^29^,^30^ To note, good provider-patient communication after diagnosis is essential so patients are well prepared for the long course of treatment. These findings align with those reported in other settings.^13^,^14^,^31^

Stigma continues to be a challenge for people affected by TB. Patients often gave incorrect contact details and went ‘missing’ due to stigma. Likewise, patients did not disclose their TB status to others, including family members. Stigma has been reported in several studies as being a reason for PTLFU.^12^–^14^,^32^ To address stigma, HCWs suggested carrying out community TB awareness campaigns and convening patient support groups. Through support groups, people affected by TB may receive money for income-generating activities to take care of basic needs and free them to concentrate on getting proper care. These suggestions support a conceptual framework in which interventions need to target the public, TB HCWs and people affected by TB to impact stigma.^33^

Furthermore, HCWs reported that there was no clear mechanism for follow-up of patients in the TB programme as seen in the HIV programme. They attributed this barrier to a lack of funds to make the calls and extra visits required to find missing patients. Studies conducted in India have reported similar findings.^14^,^15^ Follow-up with CHVs was suggested as one way to reach people affected by TB. To assist CHVs, a locator form was proposed which would provide detailed information for tracing patients. Moreover, supporting CHVs financially with a transport allowance was proposed as a motivator to make the activity sustainable.

Another barrier was the absence of pharmacists from the core TB team for decision-making. A pharmacist could help with counselling patients about medication, as well as manage supply chain challenges. Having multi-disciplinary meetings with laboratory, clinic, pharmacy and the community to review data may ensure that decisions are people-centred and lead to favourable outcomes.

To our knowledge, this is the first qualitative study in Kenya to report reasons for PTLFU. We purposively selected HCWs to ensure we captured different age groups and cadres. Furthermore, we sought views from HCWs working in TB clinic and support services, TB management and the wider community. We involved trained staff for data collection, management and analysis, and piloted our interview guide before starting data collection.

Our study had limitations. As we only interviewed HCWs from one area in Western Kenya, the findings may not apply to other areas in Kenya. Also, we did not interview national decision-makers who may have viewpoints that are not described.

## CONCLUSION

Our study highlighted multiple factors contributing to PTLFU from HCW perspectives. Although there is limited evidence to assess their impact on PTLFU, HCWs suggested practical solutions to address these factors. To effectively mitigate PTLFU, operational researchers should explore interventions aimed at addressing various levels within the healthcare ecosystem, including patients, healthcare providers and the overall healthcare system. Knowledge of TB patient management is important as it ensures the continuity of TB patient care in clinics and allows for staff rotation at the TB clinic. Offering psychosocial support to the patient throughout the care process is critical for establishing trust and correcting misperceptions about TB. By providing monthly allowance, HCWs will be able to make phone calls and physically track patients. Quarterly multi-disciplinary meetings to review TB data will be key to making informed and people-centred decisions. A qualitative study on PTLFU in Western Kenya and the rest of Kenya from the perspectives of people affected by TB should be done to complement these findings.
