# Supplementary material for: Health worker views on pre-treatment loss to follow-up in adults with pulmonary TB in Western Kenya
Source: Public Health Action. 2023 Sep 21;13(3):77–82. doi: 10.5588/pha.23.0016 (PMC10446661; doi:10.5588/pha.23.0016)
Supplement: Supplementary file 1 [file iutld_pha_23.0016_supplementarydata1.pdf]

http://dx.doi.org/10.5588/pha.23.0016

Health worker views on pre-treatment loss to follow-up in adults with pulmonary TB in Western Kenya

SUPPLEMENTARY DATA

Supplementary Data 1: Interview guide for the key informant interviews

HREC Reference No: S21/04/066(PhD) **Version: 09 June 2021**

PART A: Biodata.

**Serial code**				
**Date**				
**Age group**	**15-19**		**40-44**	
	**20-24**		**45-49**	
	**25-29**		**50-54**	
	**30-34**		**55-59**	
	**35-39**		**≥60**	
**Gender**	**Male**			
	**Female**			
**Cadre**				
**Years working in the TB clinic**				
**Years worked in TB care**				

PART B:

NOTE: To ensure the study objectives are achieved, key probes for each question will be added. The flow of the questions will be based on how the participant answers the questions

- Let's start by talking about your role in the care and management of TB patients. Can you describe to me your typical role in the care and management of TB patients?
- What are some of the challenges that you face regarding handling TB patients after the diagnosis?
- What is your understanding of the pre-treatment loss to follow-up (PTLFU)?
- What experience have you had with patients who are lost to follow-up before starting treatment?
- How would you describe the extent of PTLFU in JOOTRH?
- What are some of the factors that contribute to PTLFU?

Probe for:

  - Individual/patient-related factors
  - Provider-related factors and
  - Health systems-related factors

- What are your suggestions on what can be done to ensure we do not lose these patients before they start treatment?
- Do you have additional comments to provide regarding PTLFU?

Thank you so much for your time and your contribution to the study.

Supplementary Data 2: Informed Consent Form for participating in the key informant interview Serial Number____________ Version 01: 10 August 2021

**TITLE OF RESEARCH PROJECT:** Pre-treatment loss to follow up in adults with pulmonary tuberculosis in Kenya -contributing factors and evidence-based interventions.	
**DETAILS OF PRINCIPAL INVESTIGATOR (PI):**	
**Title, first name, surname:** Dr. Mercy Mulaku	**Ethics reference number:** S21/04/066 (PhD)
**Full postal address:** Centre for Global Health Research (CGHR_KEMRI),PO Box 1578, Kisumu, Kenya	**PI Contact number:** +254 721 559632

We would like to invite you to take part in a research project. Please take some time to read the information presented here, which will explain the details of this project. Please ask the study staff or doctor any questions about any part of this project that you do not fully understand. It is very important that you are completely satisfied that you clearly understand what this research entails and how you could be involved. Also, your participation is **entirely voluntary,** and you are free to decline to participate. In other words, you may choose to take part, or you may choose not to take part. Nothing bad will come of it if you say no: it will not affect you negatively in any way whatsoever. Refusal to participate will involve no penalty or loss of benefits or reduction in the level of care to which you are otherwise entitled. You are also free to withdraw from the study at any point, even if you do agree to take part initially.

The Health Research Ethics Committee at Stellenbosch University has approved the study. The study will be conducted according to the ethical guidelines and principles of the international Declaration of Helsinki, the South African Guidelines for Good Clinical Practice (2006), the Medical Research Council (MRC) Ethical Guidelines for Research (2002), and the Department of Health Ethics in Health Research: Principles, Processes, and Studies (2015).

What is this research study all about?

Study Site*:* This study will take place in your facility (Jaramogi Oginga Odinga Teaching and Referral Hospital). This is because it has a wider catchment area in Kisumu and other counties and most patients with tuberculosis are referred here due to the availability of diagnostics and management services.

Purpose of the study: The purpose of this study is to describe the scope of, and factors associated with pre-treatment loss to follow-up (PTLFU) and determine suggestions to reduce PTLFU in adults with pulmonary TB in Western Kenya.

Procedure: Once you permit me to participate in the study, you will participate in the interview where you will give your input on reasons for PTLFU, challenges encountered, and possible suggestions to reduce PTFLU. We plan to interview at least 12 participants, though this number might change. We may be able to interview additional participants depending on the level of saturation. The interview will be recorded if you permit me to do so, this is to transcribe later. The actual recording will be stored under lock and key, with the key only accessible to me. Although, I will also take some notes just in case the recorder fails or there is poor quality of the audio and some of the information is not clear.

Why do we invite you to participate?

We have invited you since you are part of the team that gives TB care in this hospital. Therefore, we will get valuable input regarding your experience working at the clinic.

What will your responsibilities be?

You will be required to sign the consent form whether you agree to participate or not to participate in this study. You will be asked some questions on reasons for PTLFU and challenges you face managing TB regarding PTLFU. In addition, you can also suggest to us some of the ways to address the challenges encountered.

Will you benefit from taking part in this research?

There are no financial incentives or other direct benefits to you. However, the recommendations from the discussion will be useful in improving the quality of care for adults with pulmonary TB. This will be through the identification of existing gaps and possible suggestions to reduce PTLFU to improve care.

Are there any risks involved in your taking part in this research?

This is a minimal-risk study as there will be no physical harm to you being involved in the study since it will be about us conversing regarding PTLFU. However, there may be a psychological and emotional risk during reporting sensitive issues regarding the health system challenges this will be eradicated by effective confidentiality strategies.

If you do not agree to take part, what alternatives do you have?

If you say no: it will not affect you negatively in any way whatsoever. Refusal to participate will involve no penalty or loss of benefits to which you are otherwise entitled. You are also free to withdraw from the study at any point, even if you do agree to take part initially.

Who will have access to your interview records?

All information obtained from you will be kept in confidence. The audio recorders will be kept in a locked cabinet with key access to the principal investigator only. The transcribed information will be stored in a password-protected document. At no point will your name be mentioned or used during data handling or thesis or in any resulting publications. Codes will be used instead.

Will you be paid to take part in this study and are there any costs involved?

You will be compensated to take part in the study and refreshments will be provided during the interview should you choose face to face option, since we will be conducting the interviews during your lunch break. You will not have to pay for anything if you do take part.

Is there anything else that you should know or do?

You can phone Dr. Mercy Mulaku at +254 721 559632 if you have any further queries or encounter any problems.

You can phone the Health Research Ethics Committee at 021 938 9677/9819 if there is still something that is not clear or if you have a complaint.

You will receive a copy of this information and a consent form for you to keep safe.

<table>
<tr>
<td>

Declaration by participant

By signing below, I ……………………….……………. agree to take part in a research study entitled pretreatment loss to follow up in adults with pulmonary tuberculosis in Kenya -contributing factors and evidence-based interventions.

I declare that:

- I have read this information and consent form, or it was read to me, and it is written in a language in which I am fluent and with which I am comfortable.
- I have had a chance to ask questions and I am satisfied that all my questions have been answered.
- I understand that taking part in this study is **voluntary,** and I have not been pressurized to take part.
- I know the interview will be recorded, and a transcript will be produced
- I may choose to leave the study at any time and nothing bad will come of it – I will not be penalized or prejudiced in any way.

Signed at (*place*) ....…........……………. on (*date*) …………....………..

Signature of participant …………....………...

</td>
<td>

Declaration by investigator

I *(name)* ……………………………………….……… declare that:

- I explained the information in this document simply and clearly to ………………………………….
- I encouraged him/her to ask questions and took enough time to answer them.
- I am satisfied that he/she completely understands all aspects of the research, as discussed above.
- I did/did not use an interpreter. (*If an interpreter is used then the interpreter must sign the declaration below.)*

Signed at (*place*) ....…........………. on (*date*) …………....………...

Signature of investigator…………....………...

Signature of witness…………....………...

</td>
</tr>
</table>

Signature of witness…………...………..	

Permission to have all anonymous data shared with journals:

Please carefully read the statements below (or have them read to you) and think about your choice. No matter what you decide, it will not affect whether you can be in the research study or your routine healthcare

When this study is finished, we would like to publish the results of the study in journals. Most journals require us to share your anonymous data with them before they publish the results. Therefore, we would like to obtain your permission to have your anonymous data shared with journals.

Permission for sharing samples and/or information with other investigators:

Please carefully read the statements below (or have them read to you) and think about your choice. No matter what you decide, it will not affect whether you can be in the research study or your routine health care.

To do the research, we have discussed, we must collect, and store recorded information and health information from people like you who are part of the TB care team. Once we have done the research that we are planning for this research project, we would like to store your information. Other investigators from all over the world can ask to use this recorded information in future research. To protect your privacy, we will replace your name with a unique study number. We will only use this code for information about you. We will do our best to keep the code private. It is however always possible that someone could find out about your name, but this is very unlikely to happen. Therefore, we would like to ask for your permission to share your information with other investigators.

Tick the Option you choose for anonymous data sharing with journals:

I agree to have my anonymous data shared with journals during the publication of the results of this study

☐ Signature____________

OR

I do not agree to have my anonymous data shared with journals during the publication of the results of this study

☐ Signature____________

Tick the Option you choose for sharing samples and/or information with other investigators:

I do not want my sample and/or information to be shared with other investigators

☐ Signature______________

OR

My sample and/or information may be shared with other investigators for further analysis and future research in a field related to TB research.

☐ Signature______________

Supplementary Table S1: Representative quotes on patient-related factors contributing to pre-treatment loss to follow-up.

Factor	Representative quote
Alcohol and, drug use disorders	*Q1. "We have experienced about two, I have seen two. One was from [area name], and another let me say around [area name], these alcoholic people. I don't know the fear of taking drugs." (KII_009)*
	Q2. "…. And most of them have a problem, maybe he is an alcoholic, infected, drug issues." (KII_003)
Without housing	*Q3. "In terms of the contact for easier tracing, like for example, few of our clients are homeless…." (KII_004)*
Poverty	*Q4. "I have a scenario where clients just came and told me that "I cannot start treatment; one I did not have transport to come, okay I did not have transport to reach the facility even now I don't have transport to go home." (KII_001)*
	Q5. "So, to this household, they don't have something to eat. So, when you tell her to go to the hospital and sometimes, she has reached a point where she is now weak, she can't walk. Going to the hospital becomes difficult." (KII_010)
Stigma	*Q6. "Because one time one of them was telling me when he was just here…I supplemented him but he did not have a bag to carry. So, he was just on his way boarding a matatu. So, someone said, 'So you also have this disease, you have just been supplemented.' And that one was a matatu conductor*." (KII_006)
	Q7. "…that the person is suffering from TB, you find that some don't go to the facility for treatment. They fear coming. Because when they come, they fear people will say that he is infected with HIV. Because you know any person with HIV, they say has TB. So, they still fear." (KII_010)
Misconceptions about TB	*Q8. "…like if they have TB or they have been diagnosed with TB, they think that they won't be able to carry out their normal duties, they'll stop going to work like they will be put in isolation for that period of time. And they are not comfortable when their colleagues or households are disclosed to their diagnosis." (KII_008)*
Wrong contact details	*Q9. "Maybe they have come to =JOOTRH=, I'm [name omitted], that's my name which the people in the community know me with. But the one that I have written here is not that one. Locator… I say I stay at [area name], locator, I give another. The landmark which I'm going to give is not the correct one. So, if you are going to follow up, you will circle the whole of [area name]." (KII_012)*
	Q10. "And you find that if it is NHIF, they are using somebody else's NHIF. So, they assume that person's name. We have seen such cases. When they are discharged with the result when they go back to the facility they go back with the original name. So, we are not able to track them. We have the patient; he is in the system, but we are not able to really know where he went. So, we have such cases, especially in facilities that are using NHIF." (KII_015)
Religious beliefs	*Q11. "When we come to religion, there are some denominations that say that they can't take drugs when they are sick, they will just pray and get healed." (KII_013)*
Living in a different geographical location from the study area	*Q12. "Other thing is that Kisumu County being the center of Western Kenya, facilities like =JOOTRH= where we are now tends to receive very many patients from far and wide. And that also creates a gap in that you get somebody diagnosed and that person does not come from this county, has to go back where he came from, and then we have to lose some of them in that particular way." (KII_015)*

HIV: Human Immunodeficiency Virus; **JOOTRH:** Jaramogi Oginga Odinga Teaching and Referral Hospital; NHIF: National Hospital Insurance Fund; **TB:** tuberculosis.

Supplementary Table S2: Representative quotes on healthcare system-related fctors contributing to pre-treatment loss to follow-up.

**Factor**	**Representative quote**
Lack of follow up	*Q19. "I think the system doesn't exist. Like the clinical officer will just make a phone call and say, 'this patient has not turned up today, what do we do?' We make a call, several calls, and maybe s/he is unreachable." (KII_008)*
	Q20.Then we don't have…in as much as I'm doing the contact-tracing, but we don't have a channel for…like for HIV they have people who do lost-to-follow-up. But in TB, it's hard. We don't have a channel that follows up well. (KII_002)
Drug stockouts	*Q21. "Sometimes at that point of diagnosis, whereby we have individuals that are eligible to be put on treatment. And if there is interruption in the supply of the commodity…maybe you are supposed to start today and you tell the patient, "No, come after a week?" because you are still looking for that commodity, it is not available. Some of them go and go for good. They don't come back." (KII_016)*
Long turnaround time for laboratory results	*Q22. "Just as I said, the turnaround time for our results. Like if we had a shorter…like for gene-Xpert if we could get results the same day that the patient is in the hospital, then I think we can curb that pretreatment loss to follow-up." (KII_008)*
	Q23. "So, our patient arrives in a facility in a rural setup, and the sample is collected, brought to our gene-Xpert sites, diagnosed and the result is taken back. That turn-around time of the result from the facility to the other facility, we tend to lose some patients as they are not able to be accessed." (KII_015)
Unclear referral system	*Q24. "A patient leaves a facility, and you are carrying a sample to =JOOTRH=. So, it is just to bring himself to =JOOTRH= and start the same process. So, when the result goes back to the original facility, he is not there, he already came here physically. So, we tend to kind of lose that patient." (KII_015)*
Lack of linkage from the laboratory to the clinician	*Q25. "In some facilities like the =JOOTRH=, the lab, and the TB clinic are apart. And therefore, there has to be a really clear linkage from the lab to the clinic. If there is none, we tend to lose some patients like that." (KII_015)*
	Q26. "Interdepartmental linkage sometimes is a problem. For instance, I may say from the lab, if the right information is not given, if we just send the patient to go back and pick this and come, then sometimes we end up losing that patient at the point of diagnosis. In the resulting feedback. Given from the laboratory and they walk out." (KII_016)
Lack of pharmacist involvement in meetings	*Q27. "When there is a consortium of meetings involving aspects of TB, you find that pharmacists are left out of this whole process. That has been a challenge and I think that is why we are having these issues of erratic supply of drugs and no very good management of the supply chain straight from the national government to the grassroots" (KII_019)*
Reliance on partners	*Q28. "…our sample networking process depends largely on partners. And remember partners have working time, working periods. So, when their contracts get over or rather their grants get finished, we tend to have a gap between the new grant and the old grant." (KII_015)*
Turnover of staff	*Q29. "We have had a lot of new staff coming on board and some of the old staff leaving through the support of partners. And this has gave us a gap in terms of the…knowledge gap in terms of the staff we have. So, issues like active case finding in the facility are not really effective and we need to really go back and have some form of awareness training for them." (KII_015)*

JOOTRH: Jaramogi Oginga Odinga Teaching and Referral Hospital; **TB:** tuberculosis.

Supplementary Table S3:Representative quotes on suggestions to reduce pre-treatment loss to follow-up.

Level	Suggestion	Representative quote
Provider	Psychosocial counselling and support	Q30. *"I think psychosocial counselling is very important to me just like in HIV, you see in HIV they do a thorough psychosocial counselling." (KII_001)*
		Q31. *"But you know sometimes it comes with shock and trauma. Being told that you have TB, and you look at it on the negative side, it is like a death sentence. These patients need psychological support, somebody to guide them, somebody to tell them what to do and what not to do, you see?" (KII_018)*
	Change of HCW attitude	Q32. *"And basically, we should just change our attitude and then we should just have psycho-social support. When the clients come, at least we should talk to them, and encourage them. Here you find that even when they come, we don't have that time with them." (KII_002)*
		Q33. *"If a healthcare worker creates a good rapport with that client and tries to understand the client fully and give the encouragement to that client, I think it will be making a good foundation for that client and will be able to maintain that client in terms of appointments." (KII_004)*
	Patient preparation	Q34. *"I think through…Talking to them. You take time with someone, even around 30 minutes, you tell him what TB causes, how it affects the family, how to stay…you give them some small talk. That one can…" (KII_009)*
		Q35. *"What we can do is just to empower them on TB drugs, how long TB treatment takes and time how they can take their drugs. Because this stigma starts with the individual who is the client." (KII_012)*
Healthcare system	Community Outreach	Q36. *"Maybe we can have community outreaches and health education to these patients. Because we always have them here at the waiting bay. So maybe we can always be carrying out health education around TB. we can carry out community outreaches for them to know more about TB." (KII_008)*
	Human resource and financial support	Q37. *"But still they have not put the resources in place. So, you find it's hard for us to do the contact tracing; we don't have enough resources. So maybe enough resources and enough staffing." (KII_002)*
	Having a locator form	Q38. *"We should use this locator form to actually find our client. If they are lost, we can walk out and go and look for them and bring them. Locator form will help us to locate where they are." (KII_003)*
	CHV involvement and support	Q39. "*We can bring on board and really use the community health workers who are available to really help us track patients. Once a patient has come from the community and is from Kisumu County, we are able to link that patient with the CHV who referred the patient for example. And able to follow-up…." (KII_015)*
		Q40. *"Those clients, ideally the CHVs following them should be many, and at least there should be motivation. So that those clients are followed well and get help. Because sometimes you get that most of the follow-up, we do, we do without motivation." (KII_013)*
	Patient support groups	Q41. "*But you can do it in a way that some small money is found for them to be coming in groups and then you give them some money to do their businesses…. You know to decide that the children will go without food, and you go there is a problem. You find that he is about to die but he tries to go and look for food for these children." (KII_010)*

	Access care to the nearest health facility	*Q42. "…. if they smear positive, they are supposed to be requested to go to their nearest places they come from. Because you find that some end up using a lot of transport. Now when it comes to their To Come Again (TCA) date, they tend to default. When you ask them, "I did not have transport." (*fare)". *(KII_011)*
	Results to be transmitted in real-time	*Q43. "One of the things that we are really trying…and I am really a big supporter of is we really make the sample result referral electronic. That it is real-time. That once a result is out, people are getting it in real-time and we are able to really start interventions immediately." (KII_015)*
	Interdisciplinary meetings	*Q44. "You are discussing aspects with the multi-disciplinary, let it not be just laboratory and the clinical officers. Laboratory and just the TB coordinators, you know. That brings about a gap. And then you realize most pharmacists are not aware of updates concerning treatment of TB and whatnot because they have been really left out…." (KII_019)*
		Q45. "We are trying to encourage facilities to have data review meetings. So that at the lab department for example, in that data review meeting, they are able to come up with a report of many people they saw, how many people they diagnosed positive, and these people are linked with the TB clinic, and they are outpatients." (KII_015)
	Training of staff	*Q46. "Then I would also say that we should intensify training so that we try and share with the staff issues to do with TB especially on intensive case finding, infection prevention so that they can also identify these cases and know how to manage them. I know staffing in TB, I don't know whether it is the staffing at JOOTRH which is lean, it is so lean…."(KII_001)*
	Integration of TB services with other hospital departments	*Q47. "In that, it is a department that people rotate like any other department in the hospital. Let it be integrated with other departments in the hospital fully. Then we can really see how it would work." (KII_016)*
	Effective supply of TB commodities	*Q48. "There needs to be an evaluation of aspects of improving the supply chain and making sure that all the drugs that are needed for prophylaxis and treatment of tuberculosis are available in real-time. There has to be coordination between the cadres, or the parties involved in the treatment of TB to make sure we have a therapeutic endpoint that is beneficial for the patient and the community." (KII_019)*

CHVs: Community Health Volunteers; **HIV:** Human Immunodeficiency Virus; **JOOTRH:** Jaramogi Oginga Odinga Teaching and Referral Hospital; **TB:** tuberculosis.

FIGURES

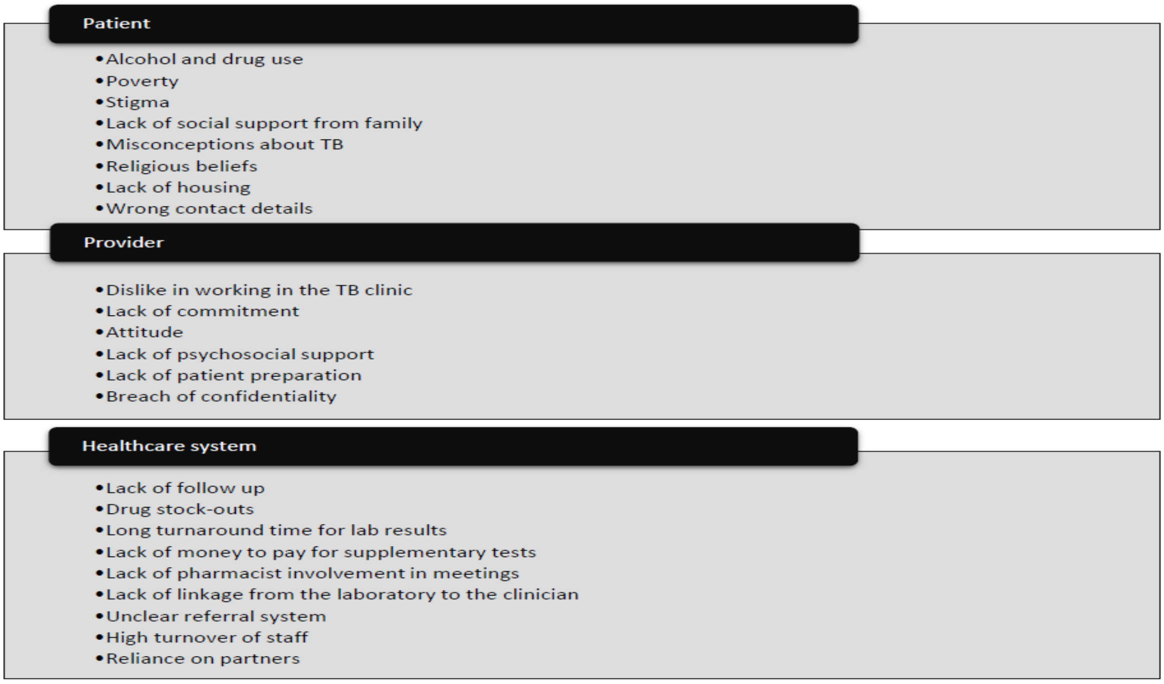

Supplementary Data 3: Factors contributing to pre-treatment loss to follow-up, perspectives of healthcare workers at JOOTRH

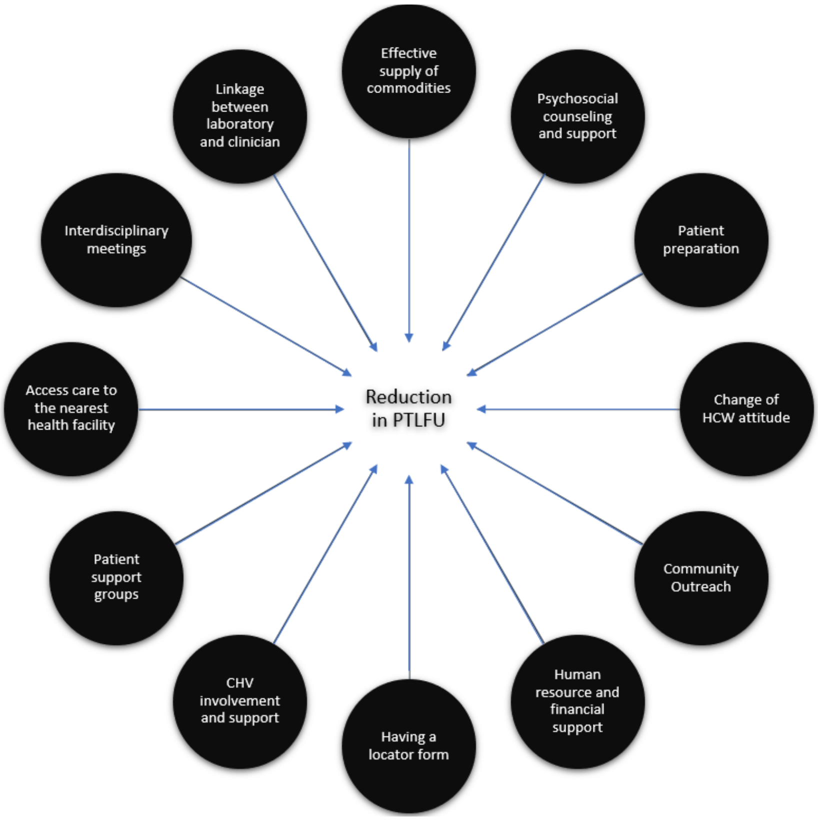

Supplementary Data 4: Suggestions to reduce pre-treatment loss to follow-up by healthcare workers at JOOTRH.
